# Vegetable peptones as a fetal bovine serum substitute in human deciduous tooth pulp stem cell culture

**DOI:** 10.31744/einstein_journal/2025AO1364

**Published:** 2025-07-11

**Authors:** Marizia Trevizani, Laís Lopardi Leal, Silvioney Augusto da Silva, Claudio Gallupo Diniz, Fabiano Freire Costa, Jair Adriano Kopke de Aguiar, Carlos Magno da Costa Maranduba

**Affiliations:** 1 Department of Biology, Institute of Biological Sciences Universidade Federal de Juiz de Fora Juiz de Fora MG Brazil Department of Biology, Institute of Biological Sciences, Universidade Federal de Juiz de Fora, Juiz de Fora, MG, Brazil.; 2 Department of Parasitology, Microbiology and Immunology Institute of Biological Sciences Universidade Federal de Juiz de Fora Juiz de Fora MG Brazil Department of Parasitology, Microbiology and Immunology, Institute of Biological Sciences, Universidade Federal de Juiz de Fora, Juiz de Fora, MG, Brazil.; 3 Department of Pharmaceutical Sciences Faculdade de Farmácia Universidade Federal de Juiz de Fora Juiz de Fora MG Brazil Department of Pharmaceutical Sciences, Faculdade de Farmácia, Universidade Federal de Juiz de Fora, Juiz de Fora, MG, Brazil.; 4 Laboratory of Glycoconjugate Analysis Institute of Biological Sciences Universidade Federal de Juiz de Fora Juiz de Fora MG Brazil Department of Biochemistry, Laboratory of Glycoconjugate Analysis, Institute of Biological Sciences, Universidade Federal de Juiz de Fora, Juiz de Fora, MG, Brazil.

**Keywords:** Peptones, Vegetables, Tooth, deciduous, Dental pulp diseases, Serum fetal , bovine, Cell- and tissue-based therapy, Steam cells, Culture

## Abstract

**Objective:**

This study aimed to evaluate the effects of different concentrations of vegetable peptones (pea, wheat, and soy) as substitutes for fetal bovine serum in stem cell cultures derived from human exfoliated deciduous teeth.

**Methods:**

Stem cell cultures derived from human exfoliated deciduous teeth were cultured with peptones in different concentrations [0.5%, 1%, and 5% (w/v)] and 10% fetal bovine serum (v/v) as control, and their proliferation was evaluated through the 3-(4,5-dimethylthiazol2-yl)-2,5-diphenyltetrazolium bromide assay. Osteogenic differentiation was assessed using Alizarin Red to quantify calcium deposition.

**Results:**

Wheat, soy, and pea concentrations greater than 1% were cytotoxic to stem cell cultures derived from human exfoliated deciduous teeth. In addition, a long-term study showed that pea peptones were cytotoxic. Studies with soy and wheat peptones were continued at concentrations of 0.5% (w/v), and proliferation on day 3 was greater than 50% compared with the control. Wheat peptone presented more mineralized areas than fetal bovine serum. The aminograms of the three peptones showed that the greater efficiency of wheat peptone may be related to its higher proline and glutamic acid proportions.

**Conclusion:**

We suggest that vegetable peptones at concentrations ≤1%, particularly 1% wheat, can be used as fetal bovine serum substitutes for stem cell cultures derived from human exfoliated deciduous teeth cultivation.

## In Brief

Fetal bovine serum is commonly avoided in cell cultures used for transplantation due to the possible contamination with antigens of animal origin. Here, we demonstrated the possibility of replacing fetal bovine serum in human deciduous dental pulp stem cell cultures with vegetable peptones. Three different peptones were tested, namely wheat, soybean, and pea.

## Highlights

■ Different vegetable peptones have different amino acid compositions.

■ Different peptones produce different crop responses.

■ Peptone composition can be compared to that of fetal bovine serum.

■ Vegetable peptones can replace fetal bovine serum.

## Graphical abstract

**Figure:**
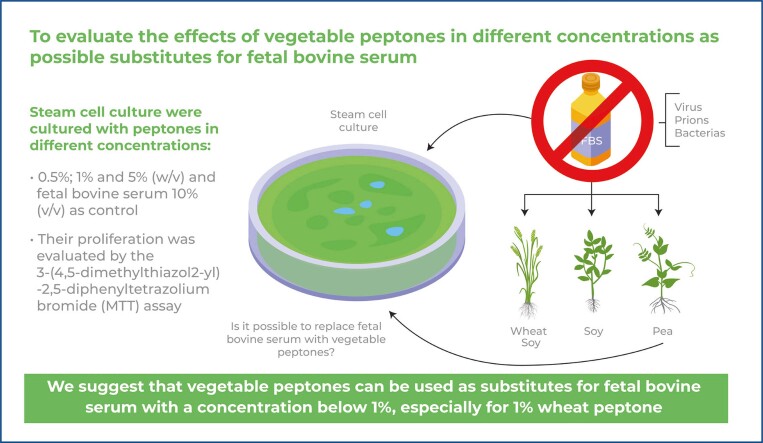

## INTRODUCTION

The success of cell therapy depends on the cell type and the efficiency of cell proliferation and plasticity.^1^ Consequently, culture media are often selectively supplemented to obtain the best *in vitro* culture parameters. Fetal bovine serum (FBS) provides a high content of growth-stimulating, nutritional, physical, and chemical factors necessary for cell maintenance and growth.^1^ Although culture medium supplemented with FBS remains the gold standard for both basic research and clinical studies, concerns have been raised owing to the potential problems associated with using FBS, including safety issues.^2^ To improve the performance of the culture medium and provide cell maintenance for long periods, the basic medium must be supplemented with other factors, such as serum. Serum supplements the culture media, stimulates the transport of phosphate, amino acids, and glucose, and increases membrane permeability. It is a protein complex involved in cell nutrition, adhesion, biological (antioxidants and antitoxins), and mechanical protection.^1^

The most commonly used serum is FBS, which contains more than one thousand different components, 200 of which are defined as vitamins, hormones, amino acids, lipids, nucleosides, transport proteins (albumin, globin, and transferrin), proliferation factors, binding factors (fibronectin and laminin), and growth factors.^3^ These substances are essential for the growth and maintenance of cells in an *in vitro* environment. However, their use in culture media can cause difficulties in the recovery and purification of bioproducts due to the presence of growth factors, proteins, and other undefined factors.^2^

In addition, the use of FBS is controversial for both ethical and scientific reasons. Fetal bovine serum is obtained from animals slaughtered in a slaughterhouse, and its composition varies according to seasonal and continental factors, which alter the serum output and make it difficult to standardize the experiments.^4^ Fetal bovine serum can also generate immunological complications, as it contains proteins that persistently stimulate human immunogenicity.^4^ Fetal bovine serum is blastogenic for peripheral blood lymphocytes,^5^ and its presence in the culture medium may be responsible for antigenic changes in the membrane of human tumor cells.^6^

Therefore, many countries defend the function and regulation of stem cells and tissue banks. In Brazil, according to (ANVISA Resolution 9/2011 - https://www.normasbrasil.com.br/norma/resolucao-9-2011_115205.html), Technology Centers for human cells and their derivatives for therapy or research and the use of products of animal origin should be avoided. However, if these products are to be used, they must be certified for the absence of infectious agents or contaminants.

## OBJECTIVE

To evaluate the effects of different concentrations of vegetable peptones (pea, wheat, and soy) on stem cell cultures derived from human exfoliated deciduous teeth.

## METHODS

### Insolation of stem cell cultures derived from human exfoliated deciduous teeth

Stem cell cultures derived from human exfoliated deciduous teeth were isolated after approval by the Human Research Ethics Committee of the *Universidade Federal de Juiz de Fora* (003/2011) and stored in the Human Genetics and Cell Therapy Biobank (CONEP 022/2015) of the Genetics Laboratory, Department of Biology, *Instituto de Ciências Biológicas, Universidade Federal de Juiz de Fora* .

### Cell cultivation

Stem cell cultures derived from human exfoliated deciduous teeth were grown at a density of 5.4 × 10^3^ cells/mL in α-minimum essential medium (Gibco- Brazil), supplemented with 1% (v/v) antibiotics (LGC Biotecnologia- Brazil), 1% (v/v) non-essential amino acids (NEAA) (Invitrogen, CA), 1% (v/v) L-glutamine (Sigma), and 10% (v/v) FBS (HyClone), in 96-well plates (CORNING, USA). The cultivation was carried out in an incubator (Incubator model RCO3000TVBB, REVCO Technologies, Asheville, USA) at 37 °C with 5% CO_2_ in atmospheric air and 95% humidity.

**Figure 1:**
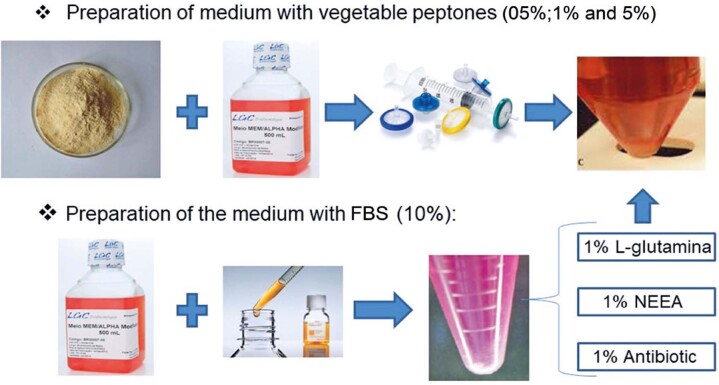
Preparation of the culture medium with peptone and fetal bovine serum FBS: fetal bovine serum.

After 24 h, the cells that adhered to the plate were viewed under a microscope to evaluate their morphology and adherence. The medium containing FBS was removed from the wells, and the medium containing vegetable peptones (peas, wheat, and soy) (Solabia Biotecnológica LTDA) was added at different concentrations [0.5%, 1%, and 5% solutions (v/v)] and supplemented with 1% (w/v) penicillin/streptomycin, 1% NEAA, and 1% L-glutamine. As an internal control, FBS was used at a standard concentration of 10% ( Figure 1 ).

### Cell viability

To evaluate cell proliferation, the 3-(4,5-dimethylthiazol2-yl)-2,5-diphenyltetrazolium bromide assay (Thiazolyl Blue Tetrazolium Blue, Sigma, USA) was performed according to the manufacturer’s protocol. Approximately 500 cells/well were plated (day 0) in a 96-well plate, and readings were taken on days 3 and 5. Cell proliferation was measured using a spectrophotometer (Varioskan Flash, Thermo Scientific, USA) at absorbances of 570, 650, and 690 nm. All experiments were performed in triplicates.

### *In vitro* osteogenic differentiation

To induce SHED differentiation, cells at an initial density of 5 × 10^3^ were seeded in 35 × 10 mm plates (Corning). The culture medium used was Dulbecco’s Modified Eagle Medium low glucose (Invitrogen), supplemented with 12% (v/v) FBS, 100 U/mL penicillin and 100 µg/mL streptomycin, 2 mM of glutamine, 0.01 mM NEAA, 0.2 mM ascorbic acid (Sigma), 10^-8^ M dexamethasone (Sigma), and 10 mM glycerophosphate (this substance was added to the medium only after the 10^th^ d). The culture was maintained for 14 d, and the medium was renewed every 3 days. After osteogenic differentiation (21 d), the culture plate was stained with Alizarin Red as described below and photographed under an inverted phase microscope (Nikon TS100F) using a 10x objective.

### Staining with Alizarin Red

To attest to osteogenic differentiation, cultures were fixed with buffered paraformaldehyde at 4% (v/v) for 24 h and stained with Alizarin Red, specific for osteogenic differentiation. The plates were washed with distilled water, and the cells were fixed in 70% ethanol for 30 min. After this period, the cells were washed once more with distilled water, and the plates were kept open until they were completely dried at room temperature. Subsequently, the plates were filled with a solution containing 1% Alizarin Red (w/v) and 1% ammonium hydroxide (v/v) in a 10:1 ratio, and kept under light agitation at room temperature for 45 min (Orbital Shaker Form, Thermo, USA). Excess stain was removed after stirring with 1x PBS, and the plates were kept open until dry. Finally, images were captured using an inverted phase microscope (Nikon TS100F, Japan) with a 10x objective.

### Quantitative analysis of calcium deposition (mineralization)

To quantify the deposition of calcium (or mineralization) in osteogenic differentiation, 250μL of extraction solution contatining 1% acetic acid and 1% methanol at a 4:1 proportion were added to each well previously stained with Alizarin Red; then, the plates were taken to the shaker (Orbital Shaker Form, Thermo, USA) for 30 min at room temperature. The content of each well was transferred to a well in a new plate, and 200μL of the extraction solution was used as a blank solution to calibrate the spectrophotometer (Thermo Scientific Varioskan Flash, USA). The calcium concentration was related to the absorbance at 495nm.

### Aminogram

The amino acid composition data for each peptone (soy, pea, and wheat) were obtained from the composition label, according to the manufacturer’s instructions (Solabia Biotecnológica LTDA). The company sent individual tables containing information on the amino acid composition (aminogram) of the peptones. From these tables, a graph was created to compare the compositions of wheat, soybean, and pea peptones.

### Statistics

Unless otherwise specified, the results are presented as means ± standard deviation (SD). Mean comparisons were performed using one-way analysis of variance, followed by Tukey’s post-hoc test. All analyses were considered statistically different when p<0.05 and were performed using GraphPad Prism version 7 (GraphPad Software, Inc.).

## RESULTS

### Effects of vegetable peptones on stem cell cultures derived from human exfoliated deciduous teeth viability and proliferation

Stem cell cultures derived from human exfoliated deciduous teeth proliferation was evaluated after replacing FBS with different concentrations of vegetable peptone for three and five days. For wheat, soy, and pea peptones, concentrations greater than 1% were cytotoxic to the cells, which made it impossible to continue the study at a concentration of 5% (data not shown). Therefore, concentrations of 0.5% and 1% were used in this study.

Concerning the viability of SHED, pea peptone showed no statistically significant difference in absorbance of cell cultures on day 3 when compared at concentrations of 0.5% and 1% (p>0.05) ( Table 1 ). However, both concentration results were significantly lower than that under 10% FBS (p<0.05), with a viability percentage of 65.69% at 0.5% and 58.64% at 1%. Likewise, the same could be observed on day 5, where the absorbances of cell cultures did not differ statistically when comparing concentrations of 0.5% and 1% of pea peptone. However, both concentrations were significantly lower than viability under 10% FBS (p<0.05), with viability percentages of 41.97% at 0.5% and 40.72% at 1%. Soy peptone showed no statistical difference between absorbances at the two concentrations applied (p>0.05) on day 3, nor when compared to the control (p>0.05). However, when the cells were exposed to five days of culture, a significant difference (p<0.05) was observed with the control for both concentrations, wherein the cell viability was 47.15% and 44.89% at concentrations of 0.5% and 1%, respectively For wheat peptone, no statistical difference was found between the absorbances (p>0.05) of cell cultures on day 3 and on day 5. However, compared with the control, the absorbances were significantly higher (p<0.05) for both concentrations applied on both days, presenting a more significant confluence. On day 3, at a concentration of 0.5%, the cell viability was 194.40% and 153.10% at a concentration of 1%. On day 5, the cell viability was 467.46% at the 0.5% concentration, and 459.14% at the 1% concentration.

**Table 1:** Cell proliferation between days 3 and 5 under exposure to different vegetable peptones

	3 days	Proliferation *versus* control (%)	FBS as 100%	5 days	Proliferation *versus* control (%)	FBS as 100%
	**Mean**	**SD**		**Mean**	**SD**	
Control (FBS)	0.123	0.011	100	0.186	0.226	100
Wheat peptone 0.5%	0.06	0.004	48.46	0.062	0.077	33.33
Wheat peptone 1%	0.072	0.011	58.24	0.062	0.064	33.33
Control (FBS)	0.064	0.062	100	0.13	0.127	100
Pea peptone 0.5%	0.044	0.039	68.75	0.087	0.1	60.77
Pea peptone 1%	0.051	0.048	79.69	0.079	0.087	60.77
Control (FBS)	0.15831	0.131744	100	0.109	0.087	100
Soy peptone 0.5%	0.062606	0.061874	39.55	0.103	0.141	94.5
Soy peptone 1%	0.093029	0.070328	58.76	0.07	0.066	64.22

FBS: fetal bovine serum.

**Figure 2:**
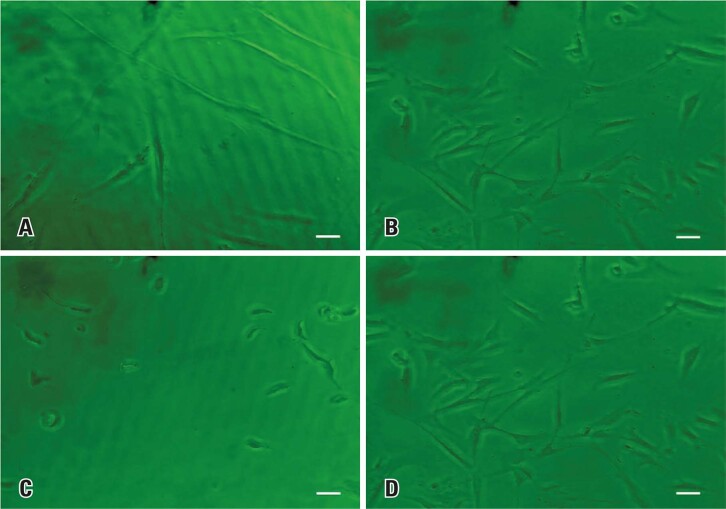
Micrographs of stem cell cultures derived from human exfoliated deciduous teeth on day 3. (A) 0.5% wheat peptone; (B) 1% wheat peptone; (C) 5% wheat peptone; (D) fetal bovine serum. Scale bar: 100 µM

As shown in figure 2 , we observed concentration-dependent morphological changes in the cell cultures supplemented with wheat peptone. Therefore, cells cultured at concentrations of 0.5% and 1% vegetable peptone maintained the typical fibroblastoid morphology of SHED. However, cells cultured with a 5% wheat peptone concentration showed a different morphology, indicating toxicity.

### Evaluation of stem cell cultures derived from human exfoliated deciduous teeth osteogenic differentiation cultivated with vegetable peptones

Concentrations of 1% for wheat and pea peptone and 0.5% for soy peptone were used to perform osteogenic differentiation. In experiments involving pea peptone, the cell culture was drastically reduced to the point where no cells adhered to the plate after 21 d of cultivation. Therefore, this experiment was discarded (data not shown).

**Figure 3:**
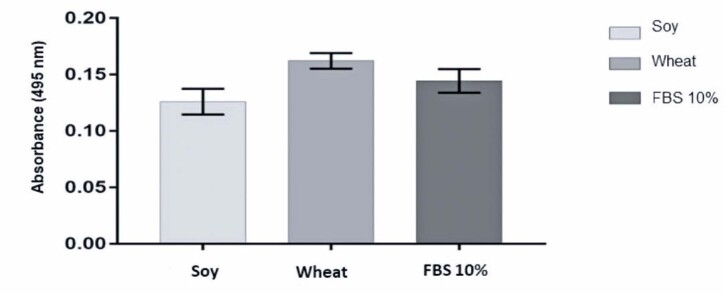
Quantification of stem cell cultures derived from human exfoliated deciduous teeth calcium deposition (mineralization) was supplemented with different vegetable peptones (0.5% soy and 1% wheat) compared with the 10% fetal bovine serum control

Wheat and soy peptones showed positive results in terms of differentiation compared to the control. When compared to FBS 10%, wheat peptone was more efficient for SHED differentiation, with 12.33% higher calcium deposits, whereas soy showed a mineralization of 87.26% ( Figure 3 ).

**Figure 4:**
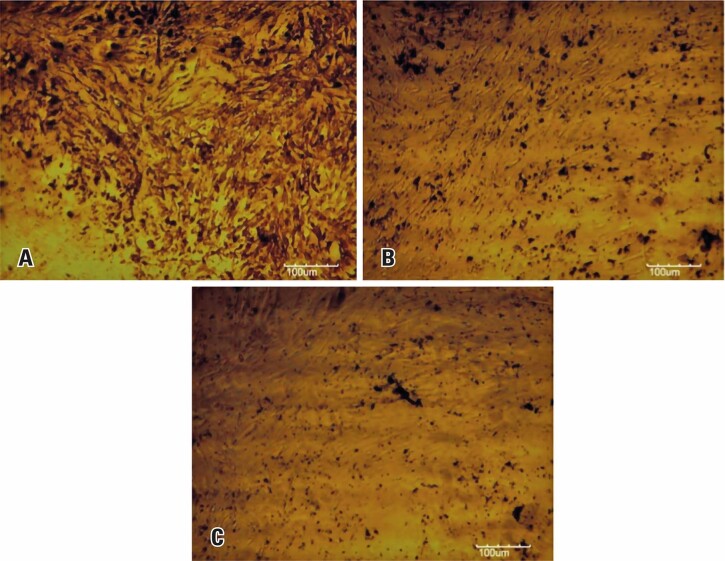
Micrographs of stem cell cultures derived from human exfoliated deciduous teeth in osteogenic differentiation. A) Soy peptone; B) wheat peptone; C) 10% fetal bovine serum (control). Scale bar: 100 µm

The results of the differentiation are shown in figure 4 , with the areas referring to the mineralization of cells induced to differentiate into osteoblasts represented in brown. The results of the differentiation showed that wheat peptone came closest to the control.

### Aminogram

**Figure 5:**
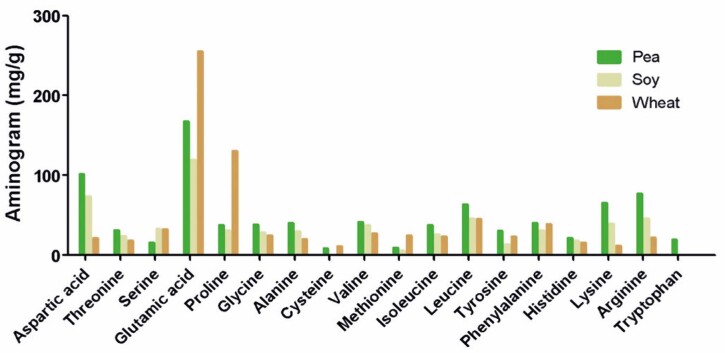
Aminogram composition of the vegetable peptones

Figure 5 shows the aminograms of the three analyzed peptones. Wheat peptone, which was more efficient than the control peptone, showed the highest glutamic acid and proline levels.

Proliferating cells metabolize glutamine via multiple bioenergetics and biosynthesis pathways.^7^ Cells can partially oxidize glutamine in a manner analogous to the partial oxidation of glucose during aerobic glycolysis.^8^ The glutaminolysis pathway contributes to the cellular production of NADH and lactate. Unlike aerobic glycolysis, glutaminolysis uses several stages of the Krebs cycle, leading to the widespread recognition that glutamine is a source of energy for proliferating cells. Thus, wheat peptone may have been the most efficient for the cultivation and differentiation of SHED because of its high glutamic acid concentration.

## DISCUSSION

The use of FBS in cell culture raises some concerns, despite containing many growth factors and extracellular matrix molecules that increase cell proliferation and differentiation. The risk of cell contamination by bovine infectious agents, such as fungi, bacteria, or viruses, and their consequent transmission to patients, as well as the possibility of cellular incorporation of animal proteins that can cause allergic reactions, increases the need to seek a potential substitute for FBS.^9^ Thus, health surveillance agencies in many countries impose the need to replace FBS in human cell cultures. FBS is available on the market, but its composition and microbiology are highly controlled. This includes Serum Hyclone^®^ (USA), which, in addition to its high cost, has a well-defined chemical composition regardless of the batch. Researchers have attempted to find a substitute for FBS, and, until now, bovine serum has remained the gold standard in terms of the efficiency of cell proliferation.^10^ Alternatives, such as serum-free media or synthetic components of plant origin, decrease the risk of contamination with infectious agents of animal origin. However, a medium without serum of animal origin must contain all extrinsic proteins necessary for adequate growth and cell proliferation.^11^

In this study, we tested the unprecedented use of soy, wheat, and pea peptones for the cultivation of SHED. We observed that the proliferation efficiency of SHED cultured for 3 and 5 days at concentrations of 0.5% and 1% varied, with wheat peptone being the most efficient among the three, and pea peptone showing a cytotoxic effect in the long term (21 d for osteogenic differentiation). Interestingly, Lee et al.^11^ demonstrated that pea and wheat peptones efficiently increased the proliferation of umbilical cord mesenchymal stem cells and those derived from adipose tissue by inducing VEGF, IL-6, and TGF-β1 production. Lundberg et al.^12^ demonstrated that concentrated pea hydrolysate generated high proliferation of mesenchymal stem cells.

In the present study, we tested the use of soy, wheat, and pea peptones for SHED cultivation in an unprecedented way. We observed that cell proliferation in 3- and 5-day cultures at concentrations of 0.5% and 1% varied, with the pea peptone being described in the literature as the most efficient for umbilical cord blood stem cells.^12^ In SHED, replacing FBS with pea peptone was the least efficient method, compared to soy and wheat peptones. Osteogenic differentiation in the presence of pea peptone resulted in cell death throughout the culture period. After 21 d of differentiation, no cells adhered to the culture plates (data not shown). The amino acids isoleucine, leucine, serine, and valine can be converted into Krebs cycle derivatives, which are indirectly involved in energy generation and directly associated with protein biosynthesis.^13^ In addition, arginine and histidine can be converted to glutamate for energy generation.^14^ Glutamate can be converted to ammonia in cellular metabolism, and the toxic effect of ammonia is evident in some cell types. The level of ammonia required to retard cell growth depends on the cell line and culture conditions. It has been suggested that ammonia accumulation disrupts the electrochemical gradient of the cell and acidifies the cytoplasm, which may lead to intracellular enzyme inactivation and induction of apoptosis.^15^ Pea peptone contains the highest quantity of amino acids that can be converted into glutamate and ammonia, leading to cell death.

Summarizing the results obtained in this study, SHED proliferation was higher when soy was used at a concentration of 0.5%, similar to the Control Group. Cell proliferation was much higher than that with 10% FBS when SHED were supplemented with wheat peptone at 0.5% or 1%. Given these results, osteogenic differentiation of SHED was induced by supplementing the medium with 0.5% soy peptone and 1% wheat peptone, followed by calcium quantification to verify mineralization. Thus, wheat peptone presented more mineralized areas and greater calcium deposition than the control, which was supplemented only with FBS. These data corroborate with previous literature, but each cell type must be tested to determine the efficiency of peptones of plant origin. Kim et al.^16^ observed that glycine promotes the growth, migration, and differentiation of steam cells through the BMP-2, Wnt, and MAPK pathways, leading to better pulp repair and regeneration. The data obtained in the present study agree with the literature,^16^ as wheat peptone contains the highest amount of glycine.

All these results could be justified through aminogram analysis, which showed that wheat peptone has a higher concentration of glutamic acid and proline. Glutamine is the primary source of skeletal muscles, which release it into the bloodstream and transported to many tissues. It is the most abundant free amino acid found in humans.^17^ Glutamine plays an essential role in promoting and maintaining the function of various organs and cells, such as the kidney,^18^ neutrophils,^19^ and heart.^20^ The intracellular concentration of glutamine varies between 2 and 20 mM depending on the cell type, whereas its extracellular concentration is 0.7 mM on average.^21^ Glutamine uptake is attributed to rapidly dividing cells such as enterocytes, fibroblasts, and lymphocytes.^22^ Glutamine is an essential precursor of peptides, proteins, purines, and pyrimidines; thus, it participates in the synthesis of nucleotides and nucleic acids.^23^

Rhoads et al.^24^ showed that glutamine activates extracellular signal-regulated kinases and c-Jun N-terminal kinases, proteins involved in signal transduction pathways stimulated by growth factors in the epithelial cell lines pig intestine-6 and pig intestine-J2, resulting in increased transcription of AP-1-dependent genes and c-Jun mRNA levels. AP-1 and c-Jun are the transcription factors that regulate gene expression during cell division. It has been postulated that glutamine enhances the effects of growth factors on cell proliferation and repair.^24^ Glutamine can stimulate the expression of adenylsuccinate synthase 1, which can regulate cell proliferation by activating protein kinase A and mTOR in neonatal rat cardiomyocytes.^25^

L-proline is an exogenous non-essential amino acid that induces the transition between mouse embryonic stem cells (ESC) and mesenchymal stem cells.^26^ This fully reversible process resembles epithelial-to-mesenchymal transition, which is essential for normal development and contributes to cancer progression. D’Aniello et al.^27^ showed that ESCs grown without L-proline have limited proliferation, which can lead to cell death. Previous studies have shown that only L-proline deprivation, among all NEAA, limits the formation of cell colonies *in vitro* .^28^ Thus, there is a strong relationship between glutamic acid and proline in cell proliferation, and a relationship can be established with the wheat peptone, which showed greater efficiency than the other analyzed peptones.

## CONCLUSION

The use of vegetable peptones as substitutes for fetal bovine serum 10% in supplementing cell culture with stem cell cultures derived from human exfoliated deciduous teeth proved to be viable since cell proliferation was equivalent (0.5% soy peptone) or higher (0.5% or 1% wheat peptone) to fetal bovine serum 10%. However, to determine the type of peptone chosen as a substitute, the amino acid composition of each vegetable peptone and the need for supplementation must be determined for each cell type. Thus, in stem cell cultures derived from human exfoliated deciduous teeth, glycine-rich supplementation is necessary for adequate cell growth and proliferation. In this sense, wheat peptone is the best alternative to using fetal bovine serum in stem cell cultures derived from human exfoliated deciduous teeth cultures, because it is rich in glutamic acid and proline. Finally, complementary studies should be carried out to evaluate the role of plant peptones as substitutes for 10% fetal bovine serum, assessing whether the peptones affect immunophenotypic characteristics, such as the expression of stem cell marker genes, as well as evaluating the cytokine profile to elucidate proliferation-inducing mechanisms.
